# The usefulness of monomeric periostin as a biomarker for idiopathic pulmonary fibrosis

**DOI:** 10.1371/journal.pone.0174547

**Published:** 2017-03-29

**Authors:** Shoichiro Ohta, Masaki Okamoto, Kiminori Fujimoto, Noriho Sakamoto, Koichiro Takahashi, Hiroshi Yamamoto, Hisako Kushima, Hiroshi Ishii, Keiichi Akasaka, Junya Ono, Ayami Kamei, Yoshinori Azuma, Hisako Matsumoto, Yukie Yamaguchi, Michiko Aihara, Takeshi Johkoh, Atsushi Kawaguchi, Masao Ichiki, Hironori Sagara, Jun-ichi Kadota, Masayuki Hanaoka, Shin-ichiro Hayashi, Shigeru Kohno, Tomoaki Hoshino, Kenji Izuhara, on behalf of the Consortium for Development of Diagnostics for Pulmonary Fibrosis Patients (CoDD-PF)

**Affiliations:** 1 Division of Medical Biochemistry, Department of Biomolecular Sciences, Saga Medical School, Saga, Japan; 2 Division of Respirology, Neurology, and Rheumatology, Department of Internal Medicine, Kurume University School of Medicine, Kurume, Japan; 3 Department of Respirology, National Hospital Organization Kyushu Medical Center, Fukuoka, Japan; 4 Department of Radiology and Center for Diagnostic Imaging, Kurume University School of Medicine, Kurume, Japan; 5 Second Department of Internal Medicine, Nagasaki University School of Medicine, Nagasaki, Japan; 6 Division of Hematology, Respiratory Medicine and Oncology, Department of Internal Medicine, Saga Medical School, Saga, Japan; 7 First Department of Internal Medicine, Shinshu University School of Medicine, Matsumoto, Japan; 8 Department of Respiratory Medicine and Infectious Diseases, Oita University Faculty of Medicine, Yufu, Japan; 9 Department of Respiratory Medicine, Dokkyo Medical University Koshigaya Hospital, Koshigaya, Japan; 10 Shino-Test Corporation, Sagamihara, Japan; 11 Department of Respiratory Medicine, Graduate School of Medicine, Kyoto University, Kyoto, Japan; 12 Department of Environmental Immuno-Dermatology, Yokohama City University Graduate School of Medicine, Yokohama, Japan; 13 Department of Radiology, Kinki Central Hospital of Mutual Aid Association of Public Teachers, Itami, Japan; 14 Center for Comprehensive Community Medicine, Saga Medical School, Saga, Japan; Medical University of South Carolina, UNITED STATES

## Abstract

The natural course of idiopathic pulmonary fibrosis (IPF) is variable. Predicting disease progression and survival in IPF is important for treatment. We previously demonstrated that serum periostin has the potential to be a prognostic biomarker for IPF. Our aim was to use monomeric periostin in a multicenter study to evaluate its efficacy in diagnosing IPF and predicting its progression. To do so, we developed a new periostin kit to detect only monomeric periostin. The subjects consisted of 60 IPF patients in a multicenter cohort study. We applied monomeric periostin, total periostin detected by a conventional kit, and the conventional biomarkers—KL-6, SP-D, and LDH—to diagnose IPF and to predict its short-term progression as estimated by short-term changes of %VC and % *D*_L, CO_. Moreover, we compared the fraction ratios of monomeric periostin to total periostin in IPF with those in other periostin-high diseases: atopic dermatitis, systemic scleroderma, and asthma. Monomeric periostin showed the greatest ability to identify IPF comparable with KL-6 and SP-D. Both monomeric and total periostin were well correlated with the decline of %VC and % *D*_L, CO_. Clustering of IPF patients into high and low periostin groups proved useful for predicting the short-term progression of IPF. Moreover, the relative ratio of monomeric periostin was higher in IPF than in other periostin-high diseases. Measuring monomeric periostin is useful for diagnosing IPF and predicting its short-term progression. Moreover, the ratio of monomeric periostin to total periostin is elevated in IPF compared to other periostin-high diseases.

## Introduction

Idiopathic pulmonary fibrosis (IPF) is a devastating disease; the median survival of IPF patients is two to three years [1]. However, the natural course of IPF is variable; some patients die within a year after diagnosis, whereas some live much longer. Predicting disease progression and survival in IPF patients is important in deciding on treatment and for prompt consideration of lung transplantation. Several pulmonary functional tests—a six-month decrease of more than 10% in FVC or 15% in *D*_L, CO_—have been shown to be useful predictors of mortality in IPF patients [2, 3]. Moreover, several serum biomarkers—surfactant proteins A and D (SP-A/SP-D, Refs. [4, 5]), KL-6 [6], and matrix metalloproteinase-7 [7]—have also been reported to be correlated with prognosis. However, if we apply the six-month decrease of FVC or *D*_L, CO_ as a biomarker to arrive at a prognosis, it takes too long when there is generally a life expectancy of only two to three years. Since most studies of serum biomarkers were performed in a single facility, a wider validation, in separate cohorts or in a multicenter analysis, has been needed. Therefore, a simple, practical, and accurate prognostic indicator in IPF is required.

Periostin is an extracellular matrix (ECM) protein belonging to the fasciclin family [8, 9]. Periostin is induced in fibroblasts by several stimuli, including TGF-β and IL-4/IL-13. We and others previously demonstrated that periostin was highly expressed in the lungs of bleomycin-administered mice or IPF patients [10–12]. Periostin expression is observed in the fibroblastic foci and adjacent to α-smooth muscle actin—positive myofibroblasts, suggesting that these are the main periostin-producing cells in pulmonary fibrosis. Periostin acts on fibroblasts together with inflammatory cytokines such as TNFα or IL-1α activating NF-κB, followed by production of various inflammatory cytokines and chemokines, leading to generation of fibrosis in the lungs [10, 13]. Thus, periostin is a key player in the pathogenesis of pulmonary fibrosis. We and others then found that serum periostin was significantly up-regulated in IPF patients [11, 12, 14]. Serum periostin was associated with six-month decreases of VC or *D*_L, CO_ [11], clinical progression [14], and overall survival and time-to-event [14]. Taken together, these results suggest the potential of periostin as a prognostic biomarker in IPF.

However, several problems have remained unresolved. First, the previous study was performed in a single facility, and the investigated number was limited. Second, it is known that serum periostin is up-regulated in patients with various inflammatory diseases other than IPF [15]. Establishment of a detection system for serum periostin more specific to IPF is needed. Although it was reported that splicing out of exon 21 is high in IPF patients, no kit to detect splicing out of exon 21 has been developed [16]. In this study, we have developed a new periostin kit specifically detecting monomeric periostin, which showed a high ratio to total periostin in IPF compared to other periostin-high diseases. We then utilized monomeric periostin in a multicenter study to evaluate its usefulness for diagnosing IPF and for predicting IPF progression.

## Materials and methods

### Antibodies

Mouse monoclonal anti-periostin antibodies SS19C, SS19D, and SS20A were established as follows. Animal studies were undertaken following the guidelines for care and use of experimental animals of the Japanese Association for Laboratory Animals Science (1987) and were approved by the Saga University Animal Care and Use Committee (Saga, Japan). Mice were intraperitoneally immunized at 2- to 4-week intervals with drosophila S2 cell-derived recombinant periostin protein emulsified in TiterMax Gold adjuvant (TiterMax USA, Norcross, GA) [17]. At least two months later, splenocytes were prepared from the mice and fused with Sp2/O myeloma cells under a standard fusion protocol using polyethylene glycol. Hybridomas producing anti-periostin IgG were selected by enzyme-linked immunosorbent assay (ELISA) with S2 recombinant periostin as an immobilized antigen. IgG was purified from culture supernatant of the hybridomas using protein G affinity chromatography. Some of the antibodies were biotinylated using EZ-Link Sulfo-NHS-LC-Biotin (Thermo Fisher Scientific, Waltham, MA), or were conjugated with horseradish peroxidase (TOYOBO, Osaka, Japan) using Sulfo-EMCS (Dojindo Laboratories, Kumamoto, Japan), according to the manufacturers’ instructions. The epitopes of SS19D and SS20A, newly generated antibodies, are the R3 domain and EMI domain, respectively, whereas the epitopes of SS18A and SS17B used for the conventional periostin ELISA are the R1 domain and the R4 domain, respectively.

### ELISA

The conventional periostin ELISA detecting total periostin (SS18A (the capture antibody)×SS17B (the detection antibody)) was previously described [11]. The new periostin ELISA was made of SS20A (the capture antibody) and SS19D (the detection antibody). Sensitivity (the limit of blank: LOB, the limit of detection: LOD, and the limit of quantification: LOQ) was evaluated according to CLSI EP-17A (National Committee for Clinical Laboratory Standards). LOB was calculated using the reproducibility of blank sample (0 ng/mL). Blank serum samples were prepared by using immunoprecipitation technique. LOD was evaluated using samples with low serum levels prepared by using immunoprecipitation. LOQ was determined with CV values obtained by intra-assay reproducibility. LOQ was defined as the lowest concentration of monomeric periostin quantifiable with CV of 10% or less.

### Preparation of monomeric periostin

Recombinant periostin proteins derived from *Drosophila* S2 cell were prepared as previously described [17], and monomeric periostin was purified with NHS-activated Sepharose beads (GE Healthcare, Little Chalfont, UK) conjugated with SS19D that was reactive to monomeric periostin (S1 Fig). Protein assay and immunoassay were used to quantify total monomeric periostin concentration. The Bradford analysis (Bio-Rad, Hercules, CA) was used for protein assay: conventional periostin ELISA (SS18A×SS17B) was used for the ELISA assay. Standard curve of the conventional periostin ELISA kit and the new periostin ELISA kit for the purified periostin protein (oligomer and monomer) and the purified monomeric periostin protein is depicted in S2 Fig.

### Immunoprecipitation and immunoblotting

Monoclonal anti-periostin antibodies were immobilized onto NHS-activated Sepharose beads at the rate of 1 mg antibody per 1 mL beads. Periostin in 0.5 mL sera was immunoprecipitated by 10 μL of SS17B-, SS18A-, SS19C-, SS19D-, or SS20A-beads, and was eluted by sample buffer containing 4% sodium dodecyl sulfate, 20% glycerol and 0.1M Tris (pH 6.8), with or without 12% 2-mercaptoethanol, followed by SDS-PAGE. Immunoblotting was essentially performed as described previously [18]. For serial immunoprecipitation, firstly periostin in 5 mL or 15 mL sera was immunoprecipitated by 10 μL of SS18A- or SS20A-beads, respectively, and the bound periostin was eluted with 0.25 M glycine (pH 2.5), followed by buffer exchange to PBS. Secondly, periostin in the eluates was immunoprecipitated by 10 μL of SS17B- or SS19D-beads, respectively, and the bound periostin was then eluted by sample buffer without 2-mercaptoethanol.

### Subjects of Idiopathic Interstitial Pneumonias (IIPs)

This study was conducted by the Consortium for Development of Diagnostics for Pulmonary Fibrosis Patients (CoDD-PF, the CoDD-PF study) composed of seven hospitals (Saga Medical School, Kurume University, Oita University, Nagasaki University, Shinshu University, National Hospital Organization Kyusyu Medical Center, and Dokkyo Medical School Koshigaya Hospital) after approval by the local institutional review boards.

The patients were enrolled from 2011 to 2014. Eligible patients with IPF and fibrotic non-specific interstitial pneumonia (fNSIP) were selected according to multidisciplinary diagnosis (MDD) following global criteria [19, 20] by two each of board-certified clinical, radiological, or histological investigators who had considerable experience in thoracic diagnosis. Concomitant therapy with up to 10 mg or the equivalent of prednisone per day was tolerated. Patients receiving other therapies for IPF, including high-dose prednisone, immunosuppressant, pirfenidone, N-acetylcysteine, and any investigational treatments for IPF, were excluded. Patients with IPF and fNSIP were eligible to participate in the present study if they were between 20 and 80 years of age and had been clinically stable with no disease exacerbation for more than 3 months prior to the first observation day. Pregnant women were excluded. Diagnosis of acute exacerbation of IPF was defined in accordance with the criteria detailed in a previous report [21].

We observed the study subjects for up to one year after the study start date (day 0). Lung functions, thin-section computed tomography (CT) images, and biomarkers were evaluated on day 0 and when the patients visited the hospitals 6–12 months after the starting date (the difference between these two times was defined as short-term change). The median (25th percentile to 75th percentile of interquartile range) of short-term duration was 189 (175–260) days. The same criteria were applied to the patients recruited for the study of biomarkers of interstitial lung disease (ILD) in the Kurume study presented in previous papers [11, 14], and the combined data from both the CoDD-PF and the Kurume studies were analyzed. The characteristics of the selected patients are described in S1 Table.

### Thin-section CT image and score interpretation

CT images were independently analyzed by two board-certified chest radiologists who were blinded to clinical information. The radiological patterns of interstitial pneumonias were as follows—definite usual interstitial pneumonia (UIP), possible UIP, or inconsistent with UIP pattern—all according to global criteria [22]. The extent of radiologic abnormalities shown in CT imaging (CT scores) were evaluated as reported previously [22, 23].

The protocol consisted of end-inspiration in the supine position, with 0.5- to 1.5-mm collimation sections reconstructed with a high-spatial-frequency algorithm at 1-cm or 2-cm intervals. Images were interpreted at a window setting appropriate for viewing the lung parenchyma (window level, -600 to -700 Hounsfield units [HU]; window width, 1200 to 1500 HU).

The radiologists evaluated the extent of the thin-section CT features, which included the presence of ground-glass attenuation, reticulation, honeycombing, emphysema, and traction bronchiectasis. The lungs were divided into six zones (upper, middle, and lower on both sides), as reported previously [22, 23]. The extents of all radiologic abnormalities were expressed as the percentage of lung parenchyma affected in each of the six zones, to the nearest 5%, and were averaged. We summed both the scores for honeycombing and reticulation into a reticular score. The scores for traction bronchiectasis were calculated by using the following formula: traction bronchiectasis score = (6 –most distal generation score) × number of segments: where the most distal generation score was obtained by assessing the generations of the most distal bronchial branches that were dilated as follows: 3, third-generation bronchi (segmental bronchi) as the main bronchus is the first generation; 4, fourth-generation bronchi (subsegmental bronchi); 5, distal to the fifth-generation bronchi; and where the number of segments was obtained by summing the number of pulmonary segments with traction bronchiectasis. The main bronchi and lobar bronchi were not counted. The range of traction bronchiectasis score that could be taken was 0 to 54.

Disagreements with respect to CT findings between the two radiologists were resolved by consensus after assessing the inter-observer agreement. Inter-observer agreements in evaluation of chest CT findings were analyzed as reported previously [11, 14]. Inter-observer agreement with regard to classification of the CT pattern of IIP was excellent (kappa, 0.87, *p*<0.001, data not shown). Assessments of the extent of CT abnormalities and traction bronchiectasis score showed a significantly high correlation between the two independent observers (Spearman *r*, 0.74–0.93, *p*<0.001, data not shown) evaluated by using Spearman’s rank correlation coefficient.

### Histological interpretation

Sixteen (10 from the CoDD-PF study and six from the Kurume study) lung specimens obtained by surgical lung biopsy (SLB) were made available for diagnosis. The histological patterns of ILD were classified as definite, probable, possible, or not UIP patterns, in accordance with global criteria [19, 20] independently by two pathologists who were blinded to clinical information. Disagreement between the two pathologists was resolved by reaching a consensus.

### Subjects of Atopic Dermatitis (AD), Systemic Scleroderma (SSc), and asthma

Serum samples were obtained from AD, SSc, and asthma patients as described previously [24–26]. SSc patients were confirmed to be free from ILD.

### Statistical analysis

Data were expressed as the mean ± standard deviation (SD). Correlations between the two parameters were evaluated using Spearman’s rank correlation coefficient. Multi-coupled comparisons were analyzed with Bonferroni correction after a Mann-Whitney U test. The cut-off levels for various parameters were defined as the optimal value for distinguishing between two groups, using a receiver operating characteristic (ROC) curve generated by logistic regression [23]. Linear regressions between variables were calculated by the least-squares method. Differences between linear regression lines were tested using analysis of covariance. All statistical analyses were performed using SPSS software (IBM SPSS Statistics, Chicago, IL) except for the ratios of monomeric periostin, for which Prism software (GraphPad Software, La Jolla, CA) was used.

## Results

### Characterization of the new periostin ELISA kit

**Fig 1:**
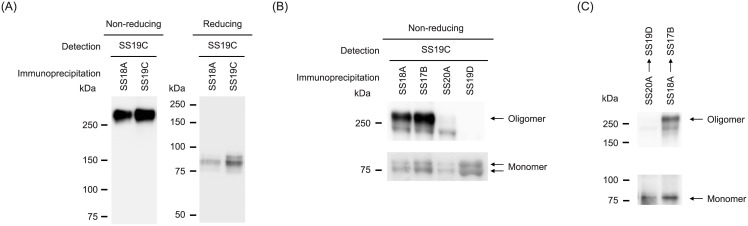
Characterization of the new periostin ELISA kit. (A) Periostin in serum was immunoprecipitated by SS18A or SS19C and detected by SS19C in non-reduced (left panel) or reduced (right panel) conditions. (B) Periostin in serum was immunoprecipitated by SS18A, SS17B, SS20A, or SS19D, respectively, and detected by SS19C in non-reduced conditions. (C) Serial immunoprecipitation of periostin by SS20A followed by SS19D (left lane) or by SS18A followed by SS17B (right lane), respectively, in non-reducing conditions.

We generated a new periostin ELISA kit composed of SS20A and SS19D among the monoclonal anti-periostin antibodies that we established. Serum periostin mostly existed in the oligomeric form, with only small amounts in the monomeric form (Fig 1A). The oligomeric form in the non-reducing condition was dissociated into the monomeric form in the reducing condition, suggesting that the oligomeric form of periostin is assembled by intramolecular disulfide bonds. We found that the newly generated monoclonal anti-periostin antibodies, SS20A and SS19D, which comprised the new periostin kit, recognized the monomeric form, but not or only faintly the oligomeric form (Fig 1B). In contrast, SS18A and SS17B, comprising the conventional periostin ELISA kit, recognized both the monomeric and oligomeric forms. Consequently, serial immunoprecipitation by SS20A, followed by SS19D mimicking the new periostin kit, precipitated only the monomeric form. But serial immunoprecipitation by SS18A, followed by SS17B mimicking the conventional periostin kit, precipitated both the monomeric and oligomeric forms (Fig 1C). The standard curves of the conventional periostin ELISA kit and the new periostin kit for the purified periostin protein (oligomer and monomer) and the purified monomeric periostin protein are shown in S2 Fig. LOB, LOD, and LOQ were estimated to be 2.7 pg/mL, 3.7 pg/mL, and 6.0 pg/mL, respectively. These results demonstrate that the new periostin kit specifically recognizes the monomeric periostin, whereas the conventional periostin kit recognizes both the monomeric and oligomeric periostin.

### Selection of patients with IPF or fNSIP

**Fig 2:**
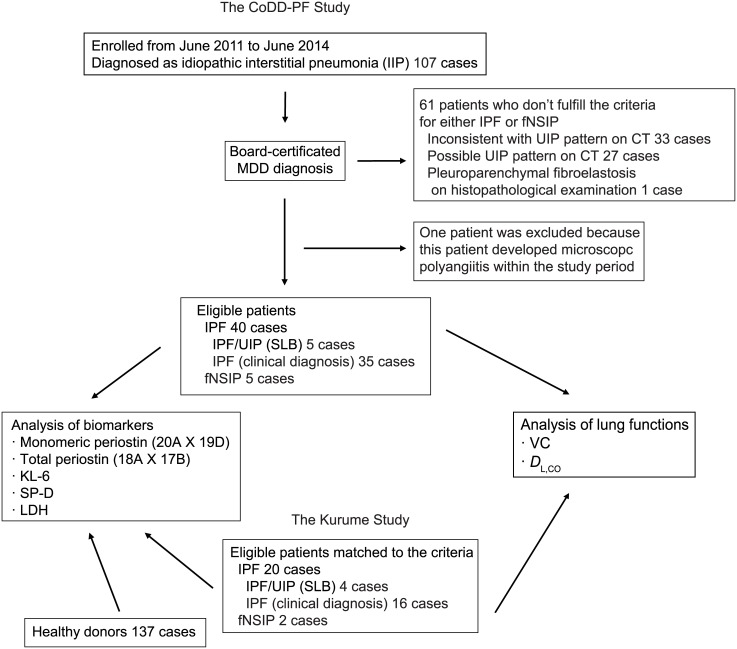
Selection of patients with IPF or fNSIP. The chart shows selection of patients with IPF or fNSIP. In the CoDD-PF study, 40 IPF patients and five fNSIP patients were selected from 107 enrolled patients. In the Kurume study, 20 IPF patients and two fNSIP patients were selected. In addition, 137 healthy donors were enrolled.

IPF and fNSIP patients who applied to participate in this study were selected as shown in Fig 2. In the CoDD-PF study, 107 patients were registered in each collaborating hospital as IIP patients, and 45 cases were selected as eligible IIP patients, comprising 40 IPF patients and five fNSIP patients, respectively. We excluded the remaining 61 cases as non-IPF patients, including one patient who developed microscopic polyangiitis during the study period. Among 40 IPF cases, five were diagnosed with UIP based on pathological examinations. Thirty-five were concordant with the clinical criteria based on the MDD by each of two experienced respiratory physicians, two thoracic radiologists, or two pathologists. All five cases of fNSIP were diagnosed by pathological examinations.

We analyzed whether pulmonary functions were correlated with the baseline or the short-term changes in the CT scores (S1 and S2 Tables). The short-term change of %*D*_L, CO_ was associated with the short-term changes of honeycombing, reticular score, or traction bronchiectasis score. These results coincide with previous reports that honeycombing, reticulation, and traction bronchiectasis score are associated with histological fibrotic process in IPF [21]. We then analyzed the correlations between %VC or %*D*_L, CO_ at the base line and at various parameters (S3 Table). Baseline % VC or %*D*_L, CO_ was weakly correlated with monomeric periostin or total periostin, respectively.

In the Kurume study, 20 IPF and two fNSIP patients fit the inclusion criteria of the CoDD-PF study. We then combined the data from both the CoDD-PF and the Kurume studies, evaluating the changes of lung functions (%VC and %*D*_L, CO_) and analyzing various biomarkers in all of the eligible patients. We also analyzed the biomarkers present in 137 healthy donors. There was no gender-based difference in monomeric or total periostin in healthy donors (monomeric periostin: male 8.6 ± 1.97 ng/mL, female 8.7 ± 2.11 ng/mL, total periostin: male 63.5 ± 18.67 ng/mL, female 67.5 ± 18.61 ng/mL).

### The greatest ability of monomeric periostin in diagnosis of IPF

**Fig 3:**
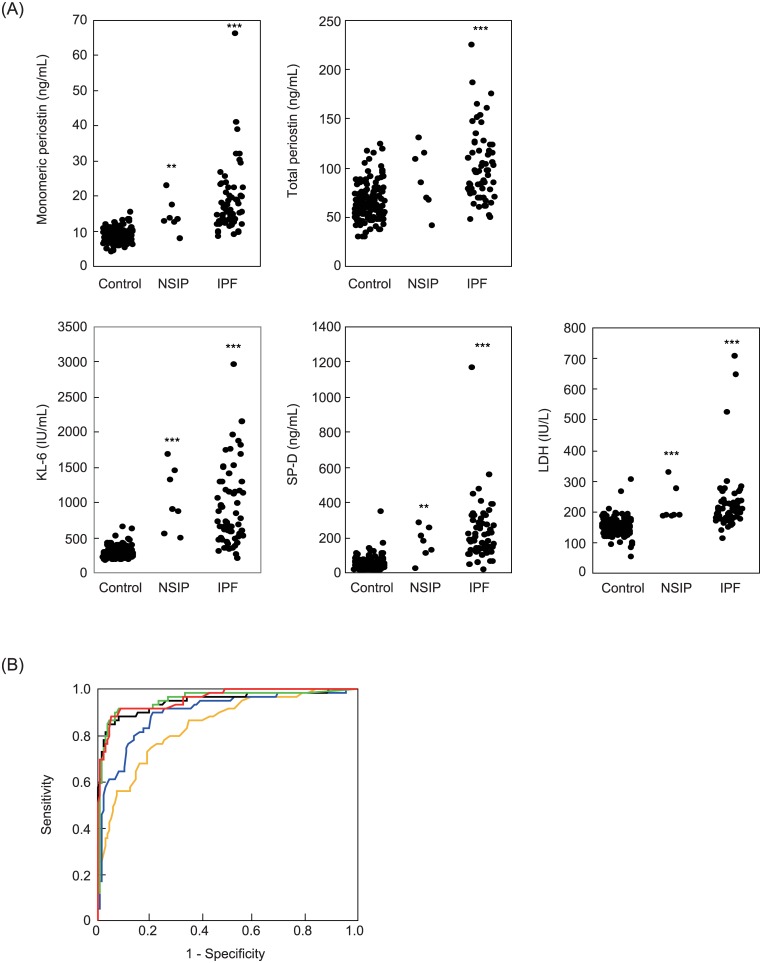
Abilities of each biomarker to diagnose IPF. (A) Serum levels of each biomarker in IPF patients, fNSIP patients, and healthy donors. Serum levels of monomeric periostin, total periostin, KL-6, SP-D, and LDH in IPF patients (n = 60), fNSIP patients (n = 7) and control donors (n = 137). (B) ROC curve analysis of each biomarker between IPF patients and healthy donors. Monomeric periostin (red), total periostin (orange), KL-6 (black), SP-D (green), and LDH (blue) between IPF patients (n = 60) and healthy donors (n = 137). ***: *p*<0.001, **: *p*<0.01.

We first measured monomeric periostin detected by the new periostin kit, total periostin detected by the conventional periostin kit, and conventional biomarkers for IPF—KL-6, SP-D, and LDH—in IPF and fNSIP patients. All of the investigated biomarkers—monomeric periostin (mean: 18.5 ± 9.5 ng/mL vs. 8.6 ± 2.0 ng/mL), total periostin (mean: 101.5 ± 36.2 ng/mL vs. 64.8 ± 18.7 ng/mL), KL-6 (mean: 932.7 ± 557.1 IU/mL vs. 289.3 ± 83.2 IU/mL), SP-D (mean: 230.2 ± 167.2 ng/mL vs. 45.8 ± 39.4 ng/mL), and LDH (mean: 225.6 ± 102.4 IU/mL vs. 150.7 ± 28.8 IU/mL)—were elevated in IPF patients (n = 60) compared to healthy donors (n = 137, *p*<0.001, Fig 3A). These biomarkers, with the exception of total periostin, were also high in fNSIP patients (n = 7, monomeric periostin: 14.3 ± 4.7 ng/mL, *p*<0.01, total periostin: 88.4 ± 31.4 ng/mL, not significant, KL-6: 1042.0 ± 454.5 IU/mL, *p*<0.001, SP-D: 170.9 ± 89.7 ng/mL, *p*<0.01, LDH: 220.7 ± 58.6 IU/mL, *p*<0.01). The distribution of monomeric periostin in healthy donors (n = 137, mean: 8.6 ± 2.0 ng/mL) is shown in S3 Fig. In the analyses with CT scores, monomeric periostin was associated with the short-term change in reticular score (S4 Table). Total periostin level was associated with the baselines of honeycombing or reticular score and the short-term change in honeycombing or reticular score. KL-6 was associated with only the baseline of reticulation. SP-D was associated with the baselines of reticulation, reticular score, or traction bronchiectasis score, and the short-term change of honeycombing.

We next performed ROC curve analyses for IPF patients (n = 60) vs. control donors (n = 137, Fig 3B). Monomeric periostin had the highest area under the curve (AUC, 0.958) among the investigated biomarkers (total periostin: 0.843, KL-6: 0.948, SP-D: 0.953, LDH: 0.898). When we set the cut-off values of monomeric periostin at 11.2 ng/mL, total periostin at 77 ng/mL, KL-6 at 398 IU/mL, SP-D at 96 ng/mL, and LDH at 166 IU/mL as the optimal points, respectively, the sensitivities and specificities were evaluated as 90.0% and 91.2% for monomeric periostin, 73.3% and 79.6% for total periostin, 88.3% and 92.0% for KL-6, 91.7% and 92.0% for SP-D, and 88.3% and 78.8% for LDH, respectively. These results demonstrate that monomeric periostin has the greatest ability to discriminate IPF patients from healthy donors among the investigated biomarkers comparable with KL-6 and SP-D.

### The abilities of monomeric and total periostin to predict short-term progression of IPF

**Fig 4:**
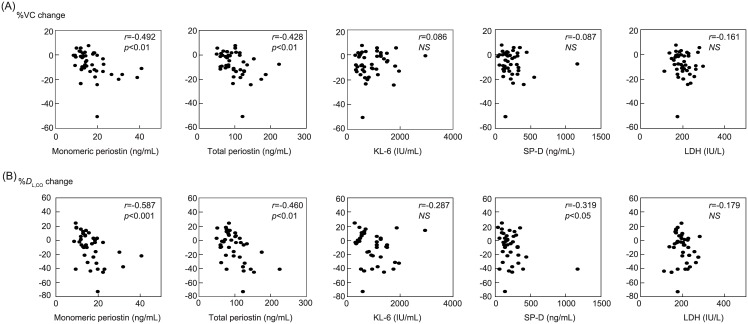
Ability of each biomarker to predict the short-term progression of IPF. Correlations between monomeric periostin, total periostin, KL-6, SP-D or LDH and short-term change of %VC (A) or %*D*_L, CO_ (B) in IPF patients (n = 44 for %VC and 39 for %*D*_L, CO_).

We next examined the correlation of each biomarker with the parameters reflecting short-term progression—short-term changes in %VC and %*D*_L, CO_—in IPF patients. Both the changes in %VC and %*D*_L, CO_ were inversely associated with monomeric periostin (*r* = −0.492, *p*<0.01 for %VC, *r* = −0.587, *p*<0.001 for %*D*_L, CO_) and total periostin (*r* = −0.428, *p*<0.01 for %VC, *r* = −0.460, *p*<0.01 for %*D*_L, CO_, Fig 4). SP-D showed a correlation with decline of %*D*_L, CO_ (*r* = −0.319, *p*<0.05), but not with %VC. Neither KL-6 nor LDH showed a significant correlation with the change of %VC or %*D*_L, CO_. It is of note that the correlations between monomeric periostin and decline of %VC were observed independently in both the CoDD-PF (*r* = −0.372, *p*<0.05) and Kurume (*r* = −0.686, *p*<0.01) studies (S4 and S5 Figs). These results show that both monomeric and total periostin are good biomarkers to predict the short-term progression of IPF.

### Usefulness of clustering IPF patients into high and low periostin groups to predict short-term progression

**Fig 5:**
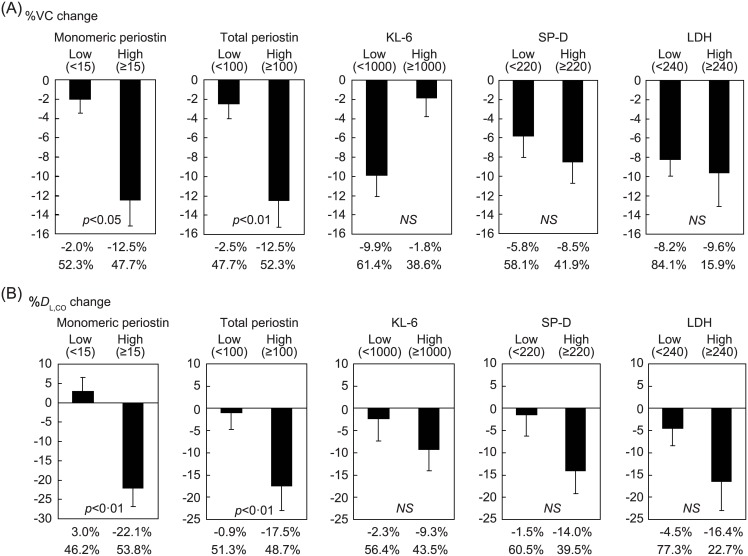
Effects of clustering IPF patients into high and low groups for each biomarker to predict the short-term progression of IPF. IPF patients were clustered into high and low groups by the cut-off values of monomeric periostin (15.0 ng/mL), total periostin (100 ng/mL), KL-6 (1,000 IU/mL), SP-D (220 ng/mL) or LDH (240 IU/L). Short-term change of %VC (A, n = 44) or %*D*_L, CO_ (B, n = 39) (upper) and proportion (down) in each high or low group is depicted.

Given that both monomeric and total periostin were well correlated with the short-term progression of IPF, we then investigated whether clustering IPF patients into high and low periostin groups would be useful to predict short-term progression. We searched for the optimal cut-off values to maximize the difference in lung function, setting the cut-off values to cluster IPF patients into high and low groups at 15.0 ng/mL for monomeric periostin, 100 ng/mL for total periostin, 1,000 IU/mL for KL-6, 220 ng/mL for SP-D, and 240 IU/mL for LDH. Then we compared the short-term changes of %VC and %*D*_L, CO_ in the high and low groups. The differences of monomeric and total periostin between the two groups were significant in both %VC (monomeric periostin: −12.5 ± 2.7% vs. −2.0 ± 1.4%, *p*<0.05, total periostin: −12.5 ± 2.7% vs. −2.5 ± 1.6%, *p*<0.01) and %*D*_L, CO_ (monomeric periostin: −22.1 ± 4.8% vs. 3.0 ± 3.6%, *p*<0.01, total periostin: −17.5 ± 5.5% vs. −0.9 ± 3.8%, *p*<0·01) changes (Fig 5). Clustering of other biomarkers did not show any significant difference between the high and low groups, even using other cut-off values. These results suggest that clustering IPF patients into high and low periostin groups is useful in predicting short-term progression.

### High ratio of monomeric periostin in IPF compared to other high-periostin diseases

**Fig 6:**
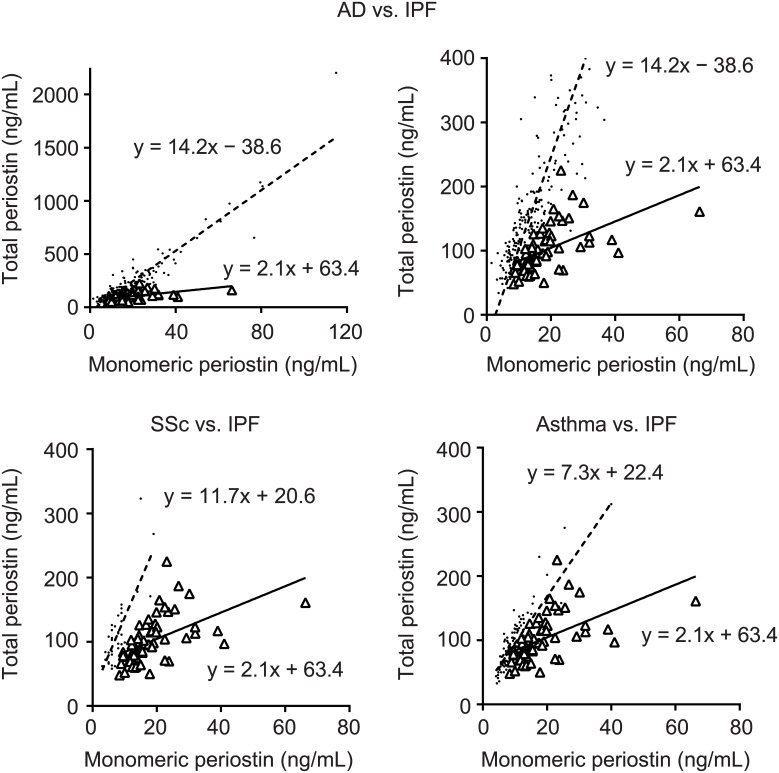
Comparison of the ratios of monomeric periostin in IPF and other high-periostin diseases. The comparison between monomeric periostin and total periostin in IPF patients (n = 60, open triangle) and AD (upper, n = 224, dot), SSc (lower left, n = 37, dot), or asthma (lower right, n = 143, dot) patients is shown. All patients or low-range patients in AD are shown in upper left and right panels, respectively. The regression lines are inserted.

We have already shown that total periostin is up-regulated in various diseases other than IPF [24–26]. Given that measuring monomeric periostin is useful in treating IPF, we compared the ratios of monomeric periostin in IPF and three other high-periostin diseases: AD, SSc, and asthma. We confirmed that the SSc patients reviewed for this study were free from ILD. The index of total periostin/monomeric periostin was significantly lower in IPF patients (2.1, n = 40) than in either AD (14.2, n = 224), SSc (11.7, n = 37), or bronchial asthma (7.3, n = 143) patients (Fig 6). These results suggest that a high ratio of monomeric periostin to total periostin is a characteristic of IPF patients.

## Discussion

We previously found that serum periostin is up-regulated in IPF patients, correlated with their decline of %VC and %*D*_L, CO_ [11]. The present study is the second prospective, independent validation cohort analysis. We have added or altered the following three points compared to the previous study: (1) we applied the new periostin ELISA kit specifically to detect the monomeric form of periostin in addition to the conventional periostin ELISA kit detecting both monomeric and oligomeric periostin (total periostin); (2) we performed this study in a consortium consisting of seven independent facilities; and (3) we compared the abilities of periostin with other conventional biomarkers for IPF—KL-6, SP-D, and LDH—in diagnosis of IPF and prediction of short-term progression of IPF. Consequently, we have revealed the following three major characteristics of monomeric periostin: (1) it has the greatest ability to diagnose IPF comparable with KL-6 and SP-D compared to total periostin; (2) it is able to predict the short-term progression of IPF comparable with total periostin; and (3) it has a high ratio to total periostin in IPF compared to other periostin-high diseases. We have confirmed these characteristics of monomeric periostin in a multi-center analysis. These results strongly support the usefulness of monomeric periostin as a diagnostic and prognostic biomarker for IPF.

We have shown that the new periostin ELISA kit, composed of SS20A×SS19D, recognizes only monomeric, but not oligomeric periostin. It is known that recombinant periostin protein prefers to form oligomers by intermolecular disulfide-bonds [27, 28]. We established that most periostin exists in oligomeric form in serum and that monomeric periostin exists as only a minor fraction. Monomeric periostin may be formed by intramolecular disulfide bonds instead of intermolecular disulfide bonds. The present study shows that production of monomeric periostin would be significantly up-regulated in IPF compared to other periostin-high diseases such as AD, SSc, and asthma. Thus far, its underlying mechanism remains unclear. It is well known that oxidative stress actively contributes to the pathogenesis of pulmonary fibrosis [29, 30]. The aberrant redox status in IPF may affect intermolecular or intramolecular disulfide-bond formation in periostin protein. It is known that periostin has eleven cysteine residues and that these cysteine residues are located at the EMI domain (Cys44, Cys60, Cys69, Cys79, Cys80, Cys92), the R1 domain (Cys208), the R2 domain (Cys311, Cys333), and the R3 domain (Cys467, Cys472), respectively. Thus far, it is unclear which cysteine residues are involved in formation of intermolecular or intramolecular disulfide-bond(s). It was previously reported that splicing out of exon 21 is high in IPF patients [16]. The correlation between production of monomeric periostin and splicing out of exon 21 is unclear, because the C-terminal domain correlated with splicing does not have any cysteine residue. However, we cannot exclude the possibility that the lack of exon 21 may affect the conformation of periostin followed by the formation of aberrant disulfide bond(s). Moreover, we did not observe different formation of oligomeric and monomeric periostin using different stimuli or cells (data not shown).

We consistently showed a good correlation of serum periostin with short-term declines of %VC and %*D*_L, CO_, which can predict mortality in IPF patients [2, 3] as reliably as reported in a previous study [11], whereas conventional biomarkers for IPF showed no or weaker correlations. Moreover, morphologic fibroproliferative findings on thin-section CT, such as reticulation and honeycombing, were also associated with monomeric or total periostin. The producing cells and the mechanism of the release of periostin or SP-D/KL-6 are likely different. Both SP-D and KL-6 are produced in regenerating alveolar type II cells; upon epithelial damage, these proteins may leak into the bloodstream [31, 32]. In contrast, myofibroblasts are likely to be the main periostin-producing cells in pulmonary fibrosis [10, 11]. Periostin expression is observed in fibroblastic foci, the sites where fibrosis is progressing [10]. These findings suggest that periostin expression would more directly reflect excess proliferation of fibroblasts or active status of fibrosis, which would lead to a decline in pulmonary functions.

Thus far, it is unknown whether oligomeric and monomeric forms of periostin have different functions. It has been reported that some specific organs such as bones or periodontal ligament [33, 34], or some cancer cells [35, 36], show different expression profiles of periostin compared to other tissues or to normal cells. Moreover, the splicing variant deleting exons 17, 18, and 21, called periodontal ligament-specific periostin or the lung type of periostin, showed enhanced binding activity to α_V_β_3_ integrin [34]. It would be possible that partner proteins in the oligomeric form of periostin may either enhance or inhibit the periostin functions. It would be of great interest to investigate whether oligomeric and monomeric forms of periostin have different functions.

There are three limitations in this study. First, in this study, we analyzed the correlation between the biomarkers and short-term changes in lung function. Although it has been shown that short-term changes in lung functions predict survival well in IPF patients [2, 3], whether monomeric periostin can predict survival as well as short-term changes in lung function needs to be addressed. Second, although in this study we increased the number of investigated subjects to 60 compared to our previous study [11], we still need to analyze a larger number of subjects in order to obtain results that are more statistically robust. Third, we did not assess sufficient numbers of other ILD patients to conclude whether the diagnostic and prognostic properties of monomeric periostin are specific for IPF. To do so, we are extending and expanding the CoDD-PF study and the Kurume study. Moreover, it is of interest to analyze whether monomeric periostin can predict the efficacy of two recently approved anti-IPF agents, pirfenidone and nintedanib [37, 38]. Although it is known that these agents are effective for some IPF patients, thus far there is no suitable biomarker to predict of efficacy of these agents. A so-called companion diagnostic for these agents would enable us to develop stratified medicine for IPF.

## Conclusions

We have developed a new periostin kit specifically to detect monomeric periostin. In a multicenter study, monomeric periostin was shown to be superior in diagnosing IPF compared to total periostin. Both monomeric and total periostin can predict short-term IPF progression better than conventional biomarkers such as KL-6, SP-D, and LDH. Moreover, the ratio of monomeric periostin to total periostin was higher in IPF compared to other periostin-high diseases. These results underscore the usefulness of measuring monomeric periostin for managing IPF.

## Supporting information

S1 Fig Preparation of total and monomeric periostin. Recombinant total periostin purified by SS18A (A) and monomeric periostin purified with SS19D (B) in non-reduced (left panel) and reduced (right panel) conditions shown in protein staining are depicted. BSA are loaded on C.
(EPS)

S2 Fig Standard curve of the conventional periostin ELISA kit and the new periostin ELISA kit for the purified periostin protein (oligomer and monomer) and the purified monomeric periostin protein. Closed and open circles represent total and monomeric periostin, respectively.
(EPS)

S3 Fig Distribution of monomeric periostin in healthy controls. Distribution of monomeric periostin detected by the new periostin ELISA kit in healthy controls (n = 137, black boxes) and IPF patients (n = 60, white boxes) is depicted.
(EPS)

S4 Fig Abilities of each biomarker to predict the short-term progression of IPF in the CoDD-PF study. Correlations between monomeric periostin, total periostin, KL-6, SP-D or LDH and the short-term change of %VC (A) or %*D*_L, CO_ (B) in IPF patients (n = 29 for %VC and 15 for %*D*_L, CO_). *NS*: not significant.
(EPS)

S5 Fig Abilities of each biomarker to predict the short-term progression of IPF in the Kurume study. Correlations between monomeric periostin, total periostin, KL-6, SP-D or LDH and the short-term change of %VC (A) or %*D*_L, CO_ (B) in IPF patients (n = 25 for %VC and 14 for %*D*_L, CO_). *NS*: not significant.
(EPS)

S1 Table Characteristics of the subjects. (DOC)

S2 Table CT findings. (DOCX)

S3 Table Correlation between baseline %VC or %*D*_L, CO_ and various parameters. (DOCX)

S4 Table Correlations between the biomarkers and the CT scores. (DOCX)
