# Supplementary material for: The usefulness of monomeric periostin as a biomarker for idiopathic pulmonary fibrosis
Source: PLoS One. 2017 Mar 29;12(3):e0174547. doi: 10.1371/journal.pone.0174547 (PMC5371347; doi:10.1371/journal.pone.0174547)
Supplement: S1 Table — (DOC) [file pone.0174547.s006.doc]

**S1 Table. Characteristics of the subjects**

|  | **IPF** | **fNSIP** | **Control** |
| --- | --- | --- | --- |
| **Number** | 60 | 7 | 137 |
| **Age** | 69.2 ± 8.1 | 66.9 ± 7.1 | 41.1 ± 11.5 |
| **Gender (Number of male)** | 55^b^ | 1 | 91 |
| **Smoking status (Number of smoker)** | 50^b^ | 2 | ND |
| **Brinkman's index (cigarettes*year)** | 758.3 ± 726.4^b^ | 261.4 ± 460.5 | ND |
| **SLB enforcement** | 9^b^ | 7 | - |
| **Term from diagnosis of IIP** | 557.2 ± 692.4 | 1598.6 ± 2840.8 | - |
| **Acute exacerbation complication** | 7 | 0 | - |
| **Serum data at baseline** |  |  |  |
| Monomeric periostin (ng/mL) | 18.5 ± 9.5 ^a^ | 14.3 ± 4.7 ^a^ | 8.6 ± 2.0 |
| Total periostin (ng/mL) | 101.5 ± 36.2 ^a^ | 88.4 ± 31.4 ^a^ | 64.8 ± 18.7 |
| KL-6 (IU/mL) | 932.7 ± 557.1 ^a^ | 1042.0 ± 454.5 ^a^ | 289.3 ± 83.2 |
| SP-D (ng/mL) | 230.2 ± 167.2 ^a^ | 170.9 ± 89.7 ^a^ | 45.8 ± 39.4 |
| LDH (IU/L) | 225.6 ± 102.4 ^a^ | 220.7 ± 58.6 ^a^ | 150.7 ± 28.8 |
| **PaO_2_ (Torr)** | 80.6 ± 14.6 | 86.5 ± 9.2 | ND |
| **Pulmonary function test at baseline** |  |  |  |
| %VC (%) | 85.8 ± 20.1 | 79.2 ± 11.7 | ND |
| % *D*_L, CO_ (%) | 59.7 ± 21.8 | 64.3 ± 15.3 | ND |
| **CT score (%) at baseline** |  |  |  |
| Ground-glass attenuation | 17.1 ± 14.3^b^ | 37.5 ± 26.7 | ND |
| Reticulation | 36.6 ± 15.7^b^ | 12.1 ± 6.4 | ND |
| Honeycombing | 10.5 ± 10.3^b^ | 0 ± 0 | ND |
| Emphysema | 14.8 ± 20.7^b^ | 1.4 ± 3.8 | ND |
| Reticular score | 47.1 ± 20.0^b^ | 12.1 ± 6.4 | ND |
| Traction bronchiectasis score | 17.7 ± 12.3 | 15.6 ± 11.4 | ND |
| **Therapy at the first observation day** |  |  |  |
| Corticosteroid | 2 | 1 | 0 |
| Long term oxygen therapy | 5 | 0 | 0 |

a: *p*<0.05 vs. control　　b: *p*<0.05 vs. fNSIP
