# Supplementary material for: The usefulness of monomeric periostin as a biomarker for idiopathic pulmonary fibrosis
Source: PLoS One. 2017 Mar 29;12(3):e0174547. doi: 10.1371/journal.pone.0174547 (PMC5371347; doi:10.1371/journal.pone.0174547)
Supplement: S2 Table — (DOCX) [file pone.0174547.s007.docx]

**S2 Table. CT findings**

|  | **Short-term change of %VC** | | | **Short-term change of %** ***D*_L, CO_** | | |
| --- | --- | --- | --- | --- | --- | --- |
|  | **n** | ***r*** | ***p* value** | **n** | ***r*** | ***p* value** |
| **Baseline** |  |  |  |  |  |  |
| Ground-glass attenuation | 44 | 0.054 | 0.727 | 39 | -0.243 | 0.137 |
| Reticulation | 44 | -0.115 | 0.456 | 39 | -0.206 | 0.209 |
| Honeycombing | 44 | -0.182 | 0.237 | 39 | -0.269 | 0.098 |
| Emphysema | 44 | 0.016 | 0.918 | 39 | 0.185 | 0.259 |
| Reticular score | 44 | -0.120 | 0.439 | 39 | -0.196 | 0.232 |
| Traction bronchiectasis score | 44 | -0.215 | 0.161 | 39 | -0.243 | 0.136 |
| **Short-term change** |  |  |  |  |  |  |
| Ground-glass attenuation | 34 | -0.060 | 0.737 | 31 | -0.031 | 0.868 |
| Reticulation | 34 | 0.058 | 0.744 | 31 | -0.108 | 0.563 |
| Honeycombing | 34 | -0.269 | 0.123 | 31 | -0.391 | 0.029* |
| Emphysema | 34 | -0.027 | 0.881 | 31 | 0.184 | 0.323 |
| Reticular score | 34 | -0.227 | 0.196 | 31 | -0.424 | 0.018* |
| Traction bronchiectasis score | 34 | -0.249 | 0.156 | 31 | -0.366 | 0.043* |

**p* values less than 0.05
