# Supplementary material for: The usefulness of monomeric periostin as a biomarker for idiopathic pulmonary fibrosis
Source: PLoS One. 2017 Mar 29;12(3):e0174547. doi: 10.1371/journal.pone.0174547 (PMC5371347; doi:10.1371/journal.pone.0174547)
Supplement: S3 Table — (DOCX) [file pone.0174547.s008.docx]

**S3 Table. Correlation between Baseline %VC or %*D*_L, CO_ and various parameters**

|  | **Baseline %VC** | | **Baseline %*D*_L, CO_** | |
| --- | --- | --- | --- | --- |
|  | **r** | ***p* value** | **r** | ***p* value** |
| **Age** | -0.12 | 0.36 | -0.036 | 0.80 |
| **Brinkman's index** | 0.13 | 0.32 | 0.12 | 0.39 |
| **Baseline data** |  |  |  |  |
| Monomeric Periositin (ng/mL) | -0.31 | 0.017* | -0.21 | 0.12 |
| Total Periositin (ng/mL) | -0.22 | 0.098 | -0.29 | 0.031 |
| KL-6 (IU/mL) | -0.15 | 0.26 | -0.32 | 0.019* |
| SP-D (ng/mL) | -0.44 | 0.0022* | -0.46 | 0.0020* |
| PaO_2_ (Torr) | 0.55 | 0.0002* | 0.69 | <0.0001* |
| **HRCTscore (%)** |  |  |  |  |
| GGA | -0.24 | 0.15 | -0.23 | 0.18 |
| Reticulation | -0.42 | 0.0081* | -0.30 | 0.071 |
| Honeycombing | -0.083 | 0.62 | -0.33 | 0.045* |
| Emphysema | 0.17 | 0.31 | 0.087 | 0.61 |
| Reticular score | -0.47 | 0.0025* | -0.41 | 0.013* |
| Traction bronchiectasis | -0.51 | 0.0010* | -0.14 | 0.40 |

**p* values less than 0.05
