# Supplementary material for: The usefulness of monomeric periostin as a biomarker for idiopathic pulmonary fibrosis
Source: PLoS One. 2017 Mar 29;12(3):e0174547. doi: 10.1371/journal.pone.0174547 (PMC5371347; doi:10.1371/journal.pone.0174547)
Supplement: S4 Table — (DOCX) [file pone.0174547.s009.docx]

**S4 Table. Correlations between the biomarkers and the CT scores**

|  | **Monomeric periostin** | | **Total periostin** | | **KL-6** | | **SP-D** | | **LDH** | |
| --- | --- | --- | --- | --- | --- | --- | --- | --- | --- | --- |
|  | ***r*** | ***p* value** | ***r*** | ***p* value** | ***r*** | ***p* value** | ***r*** | ***p* value** | ***r*** | ***p* value** |
| **Baseline** |  |  |  |  |  |  |  |  |  |  |
| Ground-glass attenuation | -0.122 | 0.352 | -0.004 | 0.979 | 0.515 | 0.085 | 0.263 | 0.042* | 0.291 | 0.025* |
| Reticulation | 0.041 | 0.753 | 0.205 | 0.116 | 0.257 | 0.048* | 0.361 | 0.005* | 0.048 | 0.717 |
| Honeycombing | 0.138 | 0.294 | 0.250 | 0.048* | 0.058 | 0.662 | 0.143 | 0.274 | -0.141 | 0.285 |
| Emphysema | -0.022 | 0.870 | -0.010 | 0.942 | -0.224 | 0.086 | -0.140 | 0.287 | -0.200 | 0.130 |
| Reticular score | 0.126 | 0.337 | 0.324 | 0.012* | 0.190 | 0.146 | 0.402 | 0.001* | 0.015 | 0.910 |
| Traction bronchiectasis score | 0.121 | 0.357 | 0.303 | 0.019 | 0.079 | 0.548 | 0.345 | 0.007* | -0.070 | 0.596 |
| **Short-term change** |  |  |  |  |  |  |  |  |  |  |
| Ground-glass attenuation | -0.164 | 0.312 | 0.052 | 0.752 | 0.063 | 0.701 | -0.010 | 0.949 | 0.106 | 0.520 |
| Reticulation | 0.180 | 0.265 | 0.190 | 0.241 | 0.080 | 0.625 | -0.290 | 0.069 | -0.108 | 0.513 |
| Honeycombing | 0.276 | 0.085 | 0.328 | 0.039* | 0.138 | 0.397 | 0.354 | 0.025* | -0.022 | 0.893 |
| Emphysema | -0.125 | 0.443 | -0.062 | 0.762 | -0.049 | 0.766 | -0.173 | 0.285 | 0.014 | 0.931 |
| Reticular score | 0.373 | 0.018* | 0.427 | 0.006* | 0.194 | 0.230 | -0.001 | 0.997 | -0.005 | 0.974 |
| Traction bronchiectasis score | 0.018 | 0.910 | 0.168 | 0.300 | 0.072 | 0.658 | -0.007 | 0.967 | 0.161 | 0.328 |

**p* values less than 0.05
